# Relationship between fatty acid intake and chronic neck/shoulder/upper limb pain without elevated CRP in a Japanese population: a cross-sectional analysis of the Shika study

**DOI:** 10.1017/jns.2022.37

**Published:** 2022-06-01

**Authors:** Atsushi Asai, Fumihiko Suzuki, Hiromasa Tsujiguchi, Akinori Hara, Sakae Miyagi, Takayuki Kannon, Keita Suzuki, Masaharu Nakamura, Yukari Shimizu, Thao Thi Thu Nguyen, Kim Oanh Pham, Tomoko Kasahara, Shingo Nakai, Koichiro Hayashi, Aki Shibata, Takashi Amatsu, Tadashi Konoshita, Yasuhiro Kambayashi, Hirohito Tsuboi, Atsushi Tajima, Hiroyuki Nakamura

**Affiliations:** 1 Department of Hygiene and Public Health, Graduate School of Medical Science, Kanazawa University, 13-1 Takaramachi, Kanazawa, Ishikawa 920-8640, Japan; 2 Department of Hygiene and Public Health, Faculty of Medicine, Institute of Medical, Pharmaceutical and Health Sciences, Kanazawa University, Kanazawa, Ishikawa 920-8640, Japan; 3 Community Medicine Support Dentistry, Ohu University Hospital, Koriyama, Fukushima 963-8611, Japan; 4 Advanced Preventive Medical Sciences Research Center, Kanazawa University, 1-13 Takaramachi, Kanazawa, Ishikawa 920-8640, Japan; 5 Innovative Clinical Research Center, Kanazawa University, 13-1 Takaramachi, Kanazawa, Ishikawa 920-8641, Japan; 6 Department of Bioinformatics and Genomics, Graduate School of Advanced Preventive Medical Sciences, Kanazawa University, 13-1 Takaramachi, Kanazawa, Ishikawa 920-8640, Japan; 7 Department of Nursing, Faculty of Health Sciences, Komatsu University, 14-1 Mukaimotorimachi, Komatsu, Ishikawa 923-0961, Japan; 8 Faculty of Public Health, Haiphong University of Medicine and Pharmacy, Ngo Quyen, Hai Phong 180000, Vietnam; 9 Third Department of Internal Medicine, University of Fukui Faculty of Medical Sciences, 23-3 Matsuoka Shimoaizuki, Eiheiji-cho, Yoshida-gun, Fukui 910-1193, Japan; 10 Department of Public Health, Faculty of Veterinary Medicine, Okayama University of Science, 1-3 Ikoinooka, Imabari, Ehime 794-8555, Japan; 11 Institute of Medical, Pharmaceutical and Health Sciences, Kanazawa University, 1 Kakuma-machi, Kanazawa 920-1192, Japan

**Keywords:** Chronic pain, C-reactive protein, Cross-sectional study, Fatty acids, Logistic model

## Abstract

Although chronic pain (CP) is classified as inflammatory or non-inflammatory, the involvement of fatty acid intake in this process has not yet been examined in detail. Therefore, the present study investigated whether the relationship between CP and fatty acid intake differs between high and low C-reactive protein (CRP) levels in middle-aged and elderly individuals in the Shika study. One-thousand and seven males and 1216 females with mean ages of 68⋅78 and 69⋅65 years, respectively, participated in the present study. CRP was quantified by blood sampling from participants who responded to a CP questionnaire. The brief-type self-administered diet history questionnaire (BDHQ) was used to assess fatty acid intake. Interactions were observed between CP and CRP on monounsaturated fatty acids (MUFA) and eicosadienoic acid in a two-way analysis of covariance adjusted for sex, age, lack of exercise, lack of sleep, current smoking and drinking status, and BMI. MUFA (OR 1⋅359) and eicosadienoic acid (OR 1⋅072) were identified as significant independent variables for CP in a multiple logistic regression analysis, but only in the low CRP group. Only a high intake of MUFA and eicosadienoic acid was associated with chronic neck/shoulder/upper limb pain without elevated CRP. In psychogenic and neuropathic pain without elevated CRP, an increased intake of MUFA and eicosadienoic acid, a family member of *n*-6 fatty acids, appears to affect CP. Further longitudinal studies are needed to elucidate this relationship.

## Graphical abstract

**Figure:**
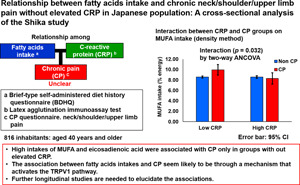

## Introduction

The prevalence of chronic pain (CP) ranges between 17⋅5 and 31⋅5 %, with variations being reported among surveyed countries and age groups^(1–4)^. A cross-sectional study by Nakamura *et al.*^(4)^ that investigated chronic musculoskeletal pain in Japanese subjects in their 30s to 50s reported that the most common pain sites were the lower back, neck, shoulders and knees. The role of inflammation in CP is considered to be greater in nociceptive pain^(5)^ and less in psychogenic^(6)^ and neuropathic pain^(7)^. Regarding the relationship between inflammation and pain, high levels of C-reactive protein (CRP) were previously shown to increase cold pain sensitivity^(8,9)^. Elevated CRP has also been reported in obesity, which increases pain sensitivity^(10,11)^. On the other hand, longitudinal studies examining the relationship between CP and CRP have found no clear relationship between chronic musculoskeletal pain and high-sensitivity CRP^(12)^. Therefore, there is currently no consensus on the relationship between CP and CRP, which warrants more detailed epidemiological studies.

Non-inflammatory factors associated with CP have been identified as professional or managerial occupations, being female, BMI > 25, and a current drinking or smoking status^(13)^. Participants with CP also cited sleep disorders, anxiety, depression or irritable bowel as possible causes^(3)^. Therefore, non-inflammatory factors need to be considered in investigations to clarify the cause of CP.

One of the non-inflammatory factors involved in CP is nutrients^(14,15)^. In our previous study^(16)^, we showed that the combination of an inadequate vitamin intake and depression was associated with CP. *n*-6 Fatty acids have also been suggested to play a role in both the induction and inhibition of pain^(17–20)^. Inflammatory cytokine production^(17)^, the involvement of complex regional pain syndrome^(18)^ and the development of mechanical allodynia^(19)^ have been implicated in pain induction. On the other hand, epoxyeicosatrienoic acid was found to inhibit pain^(20)^. However, the types of fatty acids involved in the inflammatory response, particularly CRP, have yet to be examined in detail.

Therefore, the present study investigated whether the relationship between CP and fatty acid intake differs between high and low CRP in individuals older than 40 years old in the Shika study.

## Materials and methods

### Participants

The present study was surveyed between October 2013 and January 2018. Twenty-one thousand and sixty-one people are living in Shika Town, Ishikawa Prefecture. Of these, 8499 are 65 years old or older; therefore, the aging rate is 42⋅2 %^(21)^. Participants in the present study were 5013 residents aged 40 years and older living in four model districts of Shika Town (Horimatsu, Higashimasuho, Tsuchida and Togi). Previously conducted Shika studies on CP examined CP and vitamin intake^(16)^, CP and serum 25-hydroxyvitamin D concentrations^(22)^, and the relationship between hypertension and quality of life after adjustments for CP^(23)^. Overall, 4724 participants completed the questionnaire survey and 1176 underwent a medical check-up. Participants who did not respond to the CP questionnaire, those without CRP quantification data, those who reported energy intake on the brief-type self-administered diet history questionnaire (BDHQ) was outside the range of 600–4000 kcal, and those receiving rheumatoid arthritis treatment were excluded. Therefore, 817 subjects (386 men and 431 women) were ultimately included in the analysis.

### Evaluation items

CP was defined as persistent pain for more than 3 months^(24)^ or at least twice a week in the past month. Participants were asked about the presence of CP and its location on the body using a questionnaire.

A quantitative assessment of CRP was performed on blood samples taken from participants who consented to the medical check-up. CRP was measured by the latex agglutination immunoassay test (LZ test ‘Eiken’ CRP-HG; Eiken Kagaku Company Limited, Tokyo, Japan).

BDHQ was used to investigate fatty acid intake^(25,26)^. The BDHQ is a four-page questionnaire with an average response time of 15 min. It is possible to calculate the intake of approximately thirty nutrients and fifty-eight foods using a dedicated nutrient calculation programme. The validity of BDHQ has been verified by Kobayashi *et al.*^(25,26)^. Crude data were converted using the density method, which resulted in an intake per 1000 kcal.

Participants were asked about their sex, age, BMI, lifestyle (lack of exercise, lack of sleep, and current smoking and drinking status) and treatment of underlying diseases (diabetes and dyslipidemia) using a descriptive health questionnaire.

### Statistical analysis

CP was restricted to neck/shoulder/upper limb pain and participants were classified into the non-CP and CP groups. Regarding CRP, participants were divided into low and high CRP groups based on the median of their measurements. The Student's *t* test was performed to examine relationships between continuous variables, while the *χ*^2^ test was used to investigate relationships between categorical variables. A two-way analysis of covariance (two-way ANCOVA) was employed to analyse the main effects and interactions of CP and CRP on fatty acid intake. We also examined which fatty acids were associated with CP using a multiple logistic regression analysis, stratified by high and low CRP levels, with CP as the dependent variable and fatty acid intake as the independent variable. The forced imputation method was performed to select independent variables. IBM SPSS Statistics 25 (IBM, Armonk, NY, USA) was used for statistical analyses. The significance level was set at 5 %. Of the 43 fatty acids analysed, we omitted from the table those that were not significantly different in any of the analyses. Supplementary Table S1 shows the list of fatty acids analysed in the present study.

### Sample size and statistical power

We used the free software G-power to calculate the sample size and statistical power. For the *F* tests of ANCOVA, the effect size, alpha error probability, power, number of groups and number of covariates were set to 0⋅25, 0⋅05, 0⋅95, 4 and 7, respectively. The total sample size and actual power were 210 and 0⋅950, respectively. For the *Z* tests for logistic regression, tails, odds ratio, mull hypothesis, alpha error probability, power, X distribution, X parm π were set to Two, 1⋅75, 0⋅25, 0⋅05, 0⋅95, Binomial and 0⋅5, respectively. The total sample size and actual power were found to be 791 and 0⋅950, respectively. Therefore, the sample size of this study was confirmed to be sufficient.

### Ethical standards disclosure

This study was conducted according to the guidelines laid down in the Declaration of Helsinki and all procedures involving research study participants were approved by the Ethics Committee of Kanazawa University (protocol code, 1491; date of approval, 18 December 2013). Written informed consent was obtained from all subjects involved in the study.

## Results

### Participant characteristics

Table 1 shows participant characteristics assessed for neck/shoulder/upper limb pain, CRP and fatty acid intake. Among 817 participants, 386 males had a mean age of 63⋅69 years (sd 0⋅13) and 431 females had a mean age of 63⋅82 years (sd 10⋅68), with no significant difference between the two groups. The proportion of current smokers (*P* < 0⋅001), current drinkers (*P* < 0⋅001) and diabetics (*P* = 0⋅001) were significantly higher in males than in females. BMI was also significantly higher in males than in females (*P* < 0⋅001). The intakes of saturated fatty acids (SFA) (*P* < 0⋅001), monounsaturated fatty acids (MUFA) (*P* < 0⋅001), polyunsaturated fatty acids (PUFA) (*P* < 0⋅001), *n*-3 fatty acids (*P* < 0⋅001), *n*-6 fatty acids (*P* < 0⋅001), C18:1(M) (*P* < 0⋅001), C18:2(*n*6)(P(*n*-6)) (*P* < 0⋅001), C18:3(*n*3)(P(*n*-3)) (*P* < 0⋅001) and C20:2(*n*6)(P(*n*-6)) (*P* < 0⋅001) were significantly higher in females than in males.

**Table 1.** Participant characteristics

	Male (*n* 386)		Female (*n* 431)		*P*-value*
	Mean/*n*	sd/%	Mean/*n*	sd/%	
Age, years	63⋅69	10⋅13	63⋅82	10⋅68	0⋅856
Lack of exercise, *n*	296	76⋅68	352	81⋅67	0⋅081
Lack of sleep, *n*	272	70⋅47	324	75⋅17	0⋅132
Current smoker, *n*	119	30⋅83	30	6⋅96	**<0**⋅**001**
Current drinker, *n*	286	74⋅09	98	22⋅74	**<0**⋅**001**
BMI, kg/m^2^	23⋅84	3⋅05	22⋅76	3⋅28	**<0**⋅**001**
Diabetes treatment, *n*	44	11⋅40	21	4⋅87	**0**⋅**001**
Dyslipidemia treatment, *n*	32	8⋅29	54	12⋅53	**0**⋅**047**
Neck/shoulder/upper limb pain, *n*	11	2⋅85	24	5⋅57	0⋅051
CRP, mg/dl	0⋅16	0⋅69	0⋅11	0⋅35	0⋅165
SFA, % energy	5⋅81	1⋅77	6⋅94	1⋅92	**<0**⋅**001**
MUFA, % energy	7⋅98	2⋅29	9⋅18	2⋅28	**<0**⋅**001**
PUFA, % energy	5⋅69	1⋅46	6⋅41	1⋅53	**<0**⋅**001**
*n*-3 fatty acids, % energy	1⋅27	0⋅41	1⋅40	0⋅46	**<0**⋅**001**
*n*-6 fatty acids, % energy	4⋅40	1⋅19	4⋅99	1⋅22	**<0**⋅**001**
C18:1(M), mg/1000 kcal	7861⋅77	2351⋅00	9095⋅54	2329⋅79	**<0**⋅**001**
C18:2(*n*6)(P(*n*-6)), mg/1000 kcal	4742⋅69	1298⋅81	5382⋅85	1331⋅59	**<0**⋅**001**
C18:3(*n*3)(P(*n*-3)), mg/1000 kcal	760⋅63	241⋅82	869⋅13	247⋅87	**<0**⋅**001**
C20:2(*n*6)(P(*n*-6)), mg/1000 kcal	22⋅46	8⋅08	25⋅04	8⋅66	**<0**⋅**001**

Abbreviations: sd, standard deviation; BMI, body mass index; CRP, C-reactive protein; SFA, saturated fatty acid; MUFA, monounsaturated fatty acid; PUFA, polyunsaturated fatty acid.

* *P*-values were calculated using the Student's *t* test and *χ*^2^ test for continuous and categorical variables, respectively (*P*-values < 0⋅05 are highlighted in bold).

### Comparison of CP and non-CP

The mean ages of the 35 patients in the CP (neck/shoulder/upper limb pain) group and the 782 patients in the non-CP group were 63⋅91 and 63⋅75 years, respectively, with no significant difference (Table 2). BMI was significantly higher in the non-CP group than in the CP group (*P* = 0⋅007). Fatty acid intake did not significantly differ between the two groups.

**Table 2.** Characteristics of CP and non-CP groups for fatty acid intake

	Neck/shoulder/upper limb				
	CP (*n* 35)		Non-CP (*n* 782)		*P*-value*
	Mean/*n*	95 % CI/%	Mean/*n*	95 % CI/%	
Sex (female), *n*	24	68⋅57	407	52⋅05	0⋅050
Age, years	63⋅91	60⋅11, 67⋅72	63⋅75	63⋅02, 67⋅72	0⋅927
Lack of exercise, *n*	30	85⋅71	618	79⋅03	0⋅286
Lack of sleep, *n*	29	82⋅86	567	72⋅51	0⋅128
Current smoker, *n*	7	20⋅00	141	18⋅03	0⋅770
Current drinker, *n*	15	42⋅86	369	47⋅19	0⋅616
BMI, kg/m^2^	22⋅17	21⋅42, 22⋅92	23⋅32	23⋅09, 22⋅92	**0**⋅**007**
Diabetes treatment, *n*	2	5⋅71	63	8⋅06	0⋅617
Dyslipidemia treatment, *n*	2	5⋅71	84	10⋅74	0⋅231
CRP, mg/dl	0⋅14	0⋅05, 0⋅23	0⋅14	0⋅10, 0⋅23	0⋅976
SFA, % energy	6⋅93	6⋅21, 7⋅65	6⋅38	6⋅25, 7⋅65	0⋅098
MUFA, % energy	9⋅25	8⋅34, 10⋅17	8⋅58	8⋅42, 10⋅17	0⋅100
PUFA, % energy	6⋅35	5⋅69, 7⋅00	6⋅06	5⋅95, 7⋅00	0⋅406
*n*-3 fatty acids, % energy	1⋅37	1⋅21, 1⋅53	1⋅33	1⋅30, 1⋅53	0⋅644
*n*-6 fatty acids, % energy	4⋅96	4⋅42, 5⋅49	4⋅71	4⋅62, 5⋅49	0⋅370
C18:1(M), mg/1000 kcal	9180⋅13	8248⋅03, 10 112.23	8482⋅75	8314⋅76, 10 112.23	0⋅095
C18:2(*n*6)(P(*n*-6)), mg/1000 kcal	5337⋅29	4751⋅95, 5922⋅63	5068⋅90	4975⋅5, 5922⋅63	0⋅381
C18:3(*n*3)(P(*n*-3)), mg/1000 kcal	861⋅47	752⋅05, 970⋅89	815⋅92	798⋅62, 970⋅89	0⋅426
C20:2(*n*6)(P(*n*-6)), mg/1000 kcal	25⋅75	22⋅78, 28⋅72	23⋅73	23⋅14, 28⋅72	0⋅169

Abbreviations: CI, confidence interval; BMI, body mass index; CRP, C-reactive protein; SFA, saturated fatty acid; MUFA, monounsaturated fatty acid; PUFA, polyunsaturated fatty acid.

* *P*-values were calculated using the Student's *t* test and *χ*^2^ test for continuous and categorical variables, respectively (*P*-values < 0⋅05 are highlighted in bold).

### Comparison of high and low CRP

The mean age of 453 patients in the low CRP group (62⋅71 years) was significantly lower than that of 364 patients in the high CRP group (65⋅06 years, *P* = 0⋅001) (Table 3). The proportion of females was significantly higher in the low CRP group than in the high CRP group (*P* < 0⋅001). On the other hand, the percentage of current smokers (*P* = 0⋅012), BMI (*P* < 0⋅001) and CRP quantification (*P* < 0⋅001) were significantly lower in the high CRP group than in the low CRP group. Fatty acid intake did not significantly differ between the two groups.

**Table 3.** Characteristics of low and high CRP groups for fatty acid intakes

	Low CRP (*n* 453)		High CRP (*n* 364)		*P*-value*
	Mean/*n*	95 % CI/%	Mean/*n*	95 % CI/%	
Sex (female), *n*	266	58⋅72	165	45⋅33	**<0**⋅**001**
Age, years	62⋅71	61⋅72, 63⋅70	65⋅06	64⋅05, 66⋅06	**0**⋅**001**
Lack of exercise, *n*	352	77⋅70	296	81⋅32	0⋅202
Lack of sleep, *n*	318	70⋅20	278	76⋅37	**0**⋅**047**
Current smoker, *n*	68	15⋅01	80	21⋅98	**0**⋅**012**
Current drinker, *n*	210	46⋅36	174	47⋅80	0⋅681
BMI, kg/m^2^	22⋅51	22⋅24, 22⋅77	24⋅21	23⋅87, 24⋅56	**<0**⋅**001**
Diabetes treatment, *n*	39	8⋅61	26	7⋅14	0⋅442
Dyslipidemia treatment, *n*	46	10⋅15	40	10⋅99	0⋅700
Neck/shoulder/upper limb pain, *n*	19	4⋅19	16	4⋅40	0⋅888
CRP, mg/dl	0⋅03	0⋅03, 0⋅03	0⋅27	0⋅19, 0⋅35	**<0**⋅**001**
SFA, % energy	6⋅48	6⋅31, 6⋅65	6⋅31	6⋅10, 6⋅52	0⋅207
MUFA, % energy	8⋅75	8⋅53, 8⋅97	8⋅44	8⋅20, 8⋅68	0⋅064
PUFA, % energy	6⋅15	6⋅00, 6⋅29	5⋅98	5⋅82, 6⋅13	0⋅113
*n*-3 fatty acids, % energy	1⋅34	1⋅30, 1⋅38	1⋅33	1⋅28, 1⋅37	0⋅719
*n*-6 fatty acids, % energy	4⋅79	4⋅67, 4⋅90	4⋅63	4⋅50, 4⋅75	0⋅066
C18:1(M), mg/1000 kcal	8660⋅12	8437⋅88, 8882⋅36	8329⋅08	8080⋅96, 8577⋅19	0⋅052
C18:2(*n*6)(P(*n*-6)), mg/1000 kcal	5159⋅31	5033⋅43, 5285⋅19	4982⋅19	4845⋅31, 5119⋅07	0⋅063
C18:3(*n*3)(P(*n*-3)), mg/1000 kcal	832⋅46	808⋅68, 856⋅24	799⋅71	775⋅01, 824⋅40	0⋅064
C20:2(*n*6)(P(*n*-6)), mg/1000 kcal	24⋅18	23⋅40, 24⋅95	23⋅38	22⋅49, 24⋅26	0⋅182

Abbreviations: CI, confidence interval; BMI, body mass index; CRP, C-reactive protein; SFA, saturated fatty acid; MUFA, monounsaturated fatty acid; PUFA, polyunsaturated fatty acid.

* *P*-values were calculated using the Student's *t* test and *χ*^2^ test for continuous and categorical variables, respectively (*P*-values < 0⋅05 are highlighted in bold).

### Interaction between CP and CRP on fatty acid intake

Table 4 shows the results of an analysis of the main effects and interactions of CP and CRP on fatty acid intake using a two-way ANCOVA. Covariates were adjusted for sex, age, lack of exercise, lack of sleep, current smoking or drinking status, and BMI. No fatty acids showed a main effect in the two CP groups. The fatty acids that showed a main effect in the two CRP groups were MUFA (*P* = 0⋅035), C18:1(M) (*P* = 0⋅038) and C18:3(*n*3)(P(*n*-3)) (*P* = 0⋅047). The fatty acids that showed an interaction between the CP and CRP groups were MUFA (*P* = 0⋅032), C18:1(M) (*P* = 0⋅037) and C20:2(*n*6)(P(*n*-6)) (*P* = 0⋅048). Specifically, in the high CRP group, the intakes of MUFA, C18:1(M) and C20:2(*n*6)(P(*n*-6)) were similar in the CP and non-CP groups, whereas in the low CRP group, their intakes were significantly higher in the CP group than in the non-CP group.

**Table 4.** Interaction between chronic neck/shoulder/upper limb pain and CRP on fatty acid intake

	Low CRP (*n* 453)				High CRP (*n* 364)				*P*-value*		
	Non-CP (*n* 434)		CP (*n* 19)		Non-CP (*n* 348)		CP (*n* 16)		CP	CRP	CP × CRP
	Mean	95 % CI	Mean	95 % CI	Mean	95 % CI	Mean	95 % CI			
SFA, % energy	6⋅36	6⋅19 6⋅54	7⋅17	6⋅35 7⋅98	6⋅42	6⋅22 6⋅61	6⋅51	5⋅61 7⋅40	0⋅156	0⋅337	0⋅257
MUFA, % energy	8⋅58	8⋅37 8⋅80	9⋅96	8⋅95 10⋅97	8⋅59	8⋅35 8⋅84	8⋅30	7⋅19 9⋅40	0⋅168	**0**⋅**035**	**0**⋅**032**
PUFA, % energy	6⋅06	5⋅91 6⋅20	6⋅75	6⋅08 7⋅42	6⋅07	5⋅91 6⋅23	5⋅80	5⋅07 6⋅53	0⋅415	0⋅070	0⋅063
*n*-3 fatty acids, % energy	1⋅32	1⋅28 1⋅37	1⋅44	1⋅25 1⋅64	1⋅34	1⋅30 1⋅39	1⋅27	1⋅06 1⋅48	0⋅780	0⋅318	0⋅204
*n*-6 fatty acids, % energy	4⋅71	4⋅60 4⋅82	5⋅28	4⋅75 5⋅82	4⋅70	4⋅57 4⋅83	4⋅51	3⋅92 5⋅09	0⋅358	0⋅058	0⋅064
C18:1(M), mg/1000 kcal	8485⋅79	8265⋅73 8705⋅84	9876⋅09	8846⋅26 10 905.91	8485⋅73	8237⋅71 8733⋅74	8217⋅27	7093⋅41 9341⋅12	0⋅159	**0**⋅**038**	**0**⋅**037**
C18:2(*n*6)(P(*n*-6)), mg/1000 kcal	5075⋅93	4951⋅10 5200⋅76	5690⋅18	5105⋅98 6274⋅38	5060⋅87	4920⋅17 5201⋅56	4855⋅34	4217⋅8 5492⋅88	0⋅365	0⋅061	0⋅069
C18:3(*n*3)(P(*n*-3)), mg/1000 kcal	817⋅78	794⋅38 841⋅18	932⋅57	823⋅07 1042⋅06	813⋅57	787⋅20 839⋅94	767⋅88	648⋅38 887⋅37	0⋅414	**0**⋅**047**	0⋅057
C20:2(*n*6)(P(*n*-6)), mg/1000 kcal	23⋅74	22⋅94 24⋅55	28⋅25	24⋅50 32⋅00	23⋅75	22⋅85 24⋅66	22⋅54	18⋅44 26⋅63	0⋅257	0⋅050	**0**⋅**048**

Abbreviations: CI, confidence interval; BMI, body mass index; CRP, C-reactive protein; SFA, saturated fatty acid; MUFA, monounsaturated fatty acid; PUFA, polyunsaturated fatty acid.

Adjusted for sex, age, lack of exercise, lack of sleep, current smoker, current drinker and BMI.

* A two-way analysis of covariance (*P*-values < 0⋅05 are highlighted in bold).

### Logistic regression analysis of CP on fatty acid intake stratified by CRP

Table 5 shows multiple logistic regression results stratified by high and low CRP groups, with CP as the dependent variable. Covariates were adjusted for sex, age, lack of exercise, lack of sleep, current smoking or drinking status, and BMI, with each fatty acid imputed individually into the independent variables. Significant independent variables in the low CRP group were SFA (OR 1⋅327; 95 % CI 1⋅014, 1⋅737; *P* = 0⋅040), MUFA (OR 1⋅359; 95 % CI 1⋅093, 1⋅690; *P* = 0⋅006), PUFA (OR 1⋅399; 95 % CI 1⋅033, 1⋅897; *P* = 0⋅030), *n*-6 fatty acids (OR 1⋅560; 95 % CI 1⋅068, 2⋅279; *P* = 0⋅021), C18:1(M) (OR 1⋅000; 95 % CI 1⋅000, 1⋅001; *P* = 0⋅006), C18:2(*n*6)(P(*n*-6)) (OR 1⋅000; 95 % CI 1⋅000, 1⋅001; *P* = 0⋅023), C18:3(*n*3)(P(*n*-3)) (OR 1⋅002; 95 % CI 1⋅000, 1⋅004; *P* = 0⋅028) and C20:2(*n*6)(P(*n*-6)) (OR 1⋅072; 95 % CI 1⋅015, 1⋅132; *P* = 0⋅013). In the high CRP group, no fatty acid was identified as a significant independent variable for CP. In other words, a high intake of these fatty acids significantly contributed to CP in the low CRP group only.

**Table 5.** Logistic regression analysis of CP and fatty acid intake stratified by CRP

		Exp(B)	95 % Cl Lower	96 % Cl Upper	*P*-value
Low CRP (*n* 452)	SFA, % energy	1⋅327	1⋅014	1⋅737	**0**⋅**040**
	MUFA, % energy	1⋅359	1⋅093	1⋅690	**0**⋅**006**
	PUFA, % energy	1⋅399	1⋅033	1⋅897	**0**⋅**030**
	*n*-3 fatty acids, % energy	1⋅814	0⋅675	4⋅875	0⋅238
	*n*-6 fatty acids, % energy	1⋅560	1⋅068	2⋅279	**0**⋅**021**
	C18:1(M), mg/1000 kcal	1⋅000	1⋅000	1⋅001	**0**⋅**006**
	C18:2(*n*6)(P(*n*-6)), mg/1000 kcal	1⋅000	1⋅000	1⋅001	**0**⋅**023**
	C18:3(*n*3)(P(*n*-3)), mg/1000 kcal	1⋅002	1⋅000	1⋅004	**0**⋅**028**
	C20:2(*n*6)(P(*n*-6)), mg/1000 kcal	1⋅072	1⋅015	1⋅132	**0**⋅**013**
High CRP (*n* 364)	SFA, % energy	1⋅025	0⋅790	1⋅330	0⋅853
	MUFA, % energy	0⋅926	0⋅734	1⋅170	0⋅520
	PUFA, % energy	0⋅821	0⋅561	1⋅201	0⋅309
	*n*-3 fatty acids, % energy	0⋅528	0⋅133	2⋅101	0⋅365
	*n*-6 fatty acids, % energy	0⋅799	0⋅499	1⋅279	0⋅350
	C18:1(M), mg/1000 kcal	1⋅000	1⋅000	1⋅000	0⋅552
	C18:2(*n*6)(P(*n*-6)), mg/1000 kcal	1⋅000	0⋅999	1⋅000	0⋅354
	C18:3(*n*3)(P(*n*-3)), mg/1000 kcal	0⋅999	0⋅996	1⋅001	0⋅288
	C20:2(*n*6)(P(*n*-6)), mg/1000 kcal	0⋅983	0⋅921	1⋅049	0⋅606

Abbreviations: Exp (B), Exponentiation of the B coefficient; CI, confidence interval; CRP, C-reactive protein; SFA, saturated fatty acid; MUFA, monounsaturated fatty acid; PUFA, polyunsaturated fatty acid.

Significant estimates are in bold.

## Discussion

The main result of the present study was that higher intakes of MUFA, oleic acid (C18:1) and eicosadienoic acid (C20:2) correlated with CP in the low CRP group, but not in the high CRP group.

A cross-sectional study that examined the relationship between high-sensitivity CRP and pain in daily life by Eslami *et al.*^(10)^ revealed that these two factors were related in women, but not in men; however, in a logistic regression analysis adjusted for pain-related ADL decline and BMI in women, high-sensitivity CRP was no longer associated with pain. Since the relationship between fatty acid intake and CP was influenced by CRP in the present study, this relationship appears to be complex. CRP is an indicator of inflammation^(5,27)^, which may be related to the development of CP. Therefore, high CRP is considered to be related to nociceptive pain caused by peripheral nociceptors stimulated via pain-producing substances produced through inflammation and inducing tissue damage^(5)^. The present results showing the absence of a relationship between fatty acid intake and CP in the high CRP group indicate the greater involvement of pain-producing substances during the inflammatory response. On the other hand, CP with low CRP may be explained by psychogenic pain^(6,28)^ involving depression or neuropathic pain^(29,30)^ affecting the somatosensory nervous system as pathophysiology. We speculate that a higher fatty acid intake may enhance the mechanisms involved in depression and nociceptive pathway sensitisation to CP.

*n*-6 Fatty acids, including eicosadienoic acid, are considered to play a crucial role in pain initiation and suppression^(17)^. Taken together with the involvement of the production of inflammatory eicosanoids and inflammatory cytokines in pain initiation^(17)^, elevated blood levels of *n*-6 highly unsaturated fatty acids appear to play a role in complex regional pain syndrome^(18)^ and mechanical allodynia due to the potent activation of TRPV1 in the spinal cord^(19)^. On the other hand, 14- and 15-epoxyeicosatrienoic acids have been suggested to inhibit pain^(20)^. The present results showing that the intake of eicosadienoic acid, one of the *n*-6 fatty acids, was significantly higher only in the low CRP with CP group suggested that a higher intake of *n*-6 fatty acids is related to the development of non-inflammatory psychogenic pain^(6,28)^ and low-inflammatory neuropathic pain^(29,30)^. These findings indicate that a higher intake of *n*-6 fatty acids affects non-inflammatory CP through a mechanism that activates TRPV1.

A study by Bennett and Hayes^(31)^, in which chemesthetic subqualities elicited by oleic acid (olive oil) and capsaicin were compared in healthy adults, proposed that an unknown TRPV1 agonist was present in olive oil. Therefore, a high intake of these fatty acids may exacerbate non-inflammatory psychogenic pain related to the existing quality of life^(32)^ as well as depression^(6)^ and neuropathic pain, such as trigeminal neuralgia^(7)^. The present results showed that oleic acid intake was significantly higher in the low CRP with CP group only. Therefore, a possible cause of non-inflammatory CP is the higher intake of MUFA, such as oleic acid. However, several studies^(33,34)^ indicated that a higher intake of MUFA, such as olive oil, was negatively associated with depression. On the other hand, very few epidemiological studies have investigated the relationship between MUFA and CP^(35)^. Based on previous findings, including epidemiology, the relationship between MUFA and CP remains unclear. Since the present study did not evaluate the relationship between psychogenic pain and depression, the relationships among MUFA, depression and CP need to be confirmed in longitudinal studies.

An overweight BMI as a CP-related factor has been examined in epidemiological studies^(10,11)^. A cross-sectional study that investigated elderly females without dementia revealed a relationship between obesity and pain intensity in daily life^(10)^. Similarly, a cross-sectional study on the elderly revealed a correlation between central obesity and CP^(11)^. Although these studies reported a relationship between BMI and CP, our BMI-corrected model verified that fatty acid intake correlated with CP with low CRP even after adjustments for BMI. Therefore, the present results showing a relationship between fatty acid intake and CP are not attributed to an overweight BMI.

The limitations of the present study are, first, its cross-sectional nature, due to which we were unable to examine the longitudinal causal relationship between fatty acid intake and CP. Second, since the number of participants (16 subjects) in the high CRP group with CP was small, a larger sample size analysis would be required. Third, we did not classify CRP according to reference values. Fourth, the self-administered BDHQ may lack objectivity. Fifth, we have not analysed the influence of medication treatment due to diabetes or dyslipidemia on the relationship between fatty acid intake and CP. Sixth, we have not investigated the implication of the TRPV1 pathway. Finally, since participants were volunteers, selection bias may exist.

## Conclusion

We epidemiologically revealed a relationship between fatty acid intake and CP, stratified by low and high CRP. Only high intakes of MUFA and eicosadienoic acid were associated with chronic neck/shoulder/upper limb pain without elevated CRP. In psychogenic and neuropathic pain without elevated CRP, increased intakes of MUFA and eicosadienoic acid, a family member of *n*-6 fatty acids, appear to affect CP. Further longitudinal studies are needed to elucidate this relationship.

## Supplementary material

For supplementary material accompanying this paper visit https://doi.org/10.1017/jns.2022.37. click here to view supplementary material
