# Supplementary material for: Relationship between fatty acid intake and chronic neck/shoulder/upper limb pain without elevated CRP in a Japanese population: a cross-sectional analysis of the Shika study
Source: J Nutr Sci. 2022 Jun 1;11:e38. doi: 10.1017/jns.2022.37 (PMC9161036; doi:10.1017/jns.2022.37)
Supplement: Supplementary file 1 [file S2048679022000374sup001.docx]

Table S1 List of fatty acids evaluated in the present study

| C4:0(S) |
| --- |
| C6:0(S) |
| C8:0(S) |
| C10:0(S) |
| C10:1(M) |
| C12:0(S) |
| C14:0(S) |
| C14:1(M) |
| C15:0(S) |
| C16:0(S) |
| C16:1(M) |
| C16:3(?)(P(n-6)) |
| C17:0(S) |
| C17:1(M) |
| C18:0(S) |
| C18:1(M) |
| C18:2(n6)(P(n-6)) |
| C18:3(n3)(P(n-3)) |
| C18:3(n6)(P(n-6)) |
| C18:4(n3)(P(n-3)) |
| C20:0(S) |
| C20:1(M) |
| C20:2(n6)(P(n-6)) |
| C20:3(n6)(P(n-6)) |
| C20:4(n3)(P(n-3)) |
| C20:4(n6)(P(n-6)) |
| C20:5(n3)(P(n-3)) |
| C22:0(S) |
| C22:1(M) |
| C22:5(n3)(P(n-3)) |
| C22:5(n6)(P(n-6)) |
| C22:6(n3)(P(n-3)) |
| C24:0(S) |
| C24:1(M) |
| C7_0 |
| C13_0 |
| C15_0A |
| C16_0I |
| C17_0A |
| C16_2 |
| C16_4 |
| C21_5N3 |
| C22_4N6 |
